# Screening and functional analysis of a host membrane protein interacting with the structural protein VP1 of deformed wing virus

**DOI:** 10.3389/fmicb.2026.1860293

**Published:** 2026-08-05

**Authors:** Di Liu, Yuting Xiao, Xu Li, Hanwen Zhang, Yue Zhang, Guan Wang, Xuechen Yin, Yang Yu, Haiyu Liu, Yuming Liu, Li Sun, Ming Li, Yonghua Liu, Ma Mingxiao, Dongliang Fei

**Affiliations:** 1 Collaborative Innovation Center for Zoonosis Prevention and Treatment, Jinzhou Medical University, Jinzhou, China; 2 Key Laboratory of Livestock Products Quality and Safety Engineering of Liaoning Province, Jinzhou Medical University, Jinzhou, China; 3 Tianjin Challenge Biotechnology Co., Ltd., Tianjin, China; 4 College of Animal Science and Veterinary Medicine, Shenyang Agricultural University, Shenyang, China

**Keywords:** deformed wing virus, interacting proteins, viral replication, VP1 protein, yeast membrane protein two-hybrid system

## Abstract

**Introduction:**

Deformed wing virus (DWV) is a major viral pathogen infecting Western honeybees (*Apis mellifera*). However, the interactions between viral structural proteins and host proteins during infection remain poorly understood. In this study, we used a yeast membrane protein two-hybrid system to screen for host membrane proteins that interact with the DWV structural protein VP1. The identified interactions were further functionally characterized using glutathione S-transferase (GST) pull-down, co-immunoprecipitation (Co-IP) and RNA interference (RNAi) assays.

**Methods:**

The bait plasmid pBT3-STE-VP1, carrying the VP1 gene, was screened against a complementary DNA library of Western honeybee proteins. Among the 22 candidate host proteins identified, a neuropeptide capa (NCR) like G-protein-coupled receptor was selected for further analysis. The interaction between DWV VP1 and NCR-like was confirmed using GST pull-down assays, Co-IP and co-expression. Using healthy bee pupae as experimental subjects, RNAi mediated knockdown of the NCR-like gene was performed to investigate its effect on the expression of antimicrobial peptides (AMPs), including Defensin-1 and Hymenoptaecin. Furthermore, RNAi was used to silence the NCR-like gene in DWV-infected bee pupae to evaluate its impact on viral replication.

**Results:**

Viral replication levels increased approximately sixfold compared with the infection-only control group. These results indicate that DWV VP1 interacts with the host NCR-like protein to regulate the expression of immune effectors, particularly AMPs, thereby influencing DWV replication.

**Discussion:**

This study provides initial mechanistic insights into interactions between DWV and its host, as well as the viral pathogenic mechanisms.

## 1 Introduction

Honeybees (*Apis mellifera*) play an irreplaceable role in maintaining plant biodiversity and ensuring stable and enhanced crop yields (Genersch, 2010). Recently, however, recently, the health of honeybee colonies has been increasingly threatened by various pathogens (Nazzi et al., 2012). Among these, deformed wing virus (DWV) is the most prevalent and damaging pathogens worldwide (Chen et al., 2021). In addition to shortening the lifespan of bees, DWV contributes to colony collapse disorder (CCD) (Parekh et al., 2022) and overwintering colony losses. Consequently, this pathogen severely compromises the sustainable development of the beekeeping industry, leading to significant economic losses (De Miranda and Genersch, 2010).

DWV is a single-stranded, positive-sense RNA virus belonging to the genus Iflavirus within the family Iflaviridae order Picornavirales. The viral particles have a non-enveloped icosahedral structure with a diameter of approximately 30 nm (Fei et al., 2019). The DWV genome is approximately 10,000 bp in length and contains a single large open reading frame encoding a polyprotein precursor of 2,894 amino acids. Following proteolytic cleavage of this precursor, three nonstructural proteins (an RNA helicase, a 3C protease, and an RNA-dependent RNA polymerase) and three major structural proteins VP1, VP2, and VP3 are generated (Organtini et al., 2017; Sun et al., 2023; Sui et al., 2015). Among these, the capsid protein VP1 has the highest molecular weight (44 kDa), which is significantly larger than that of VP2 (~32 kDa) and VP3 (~28 kDa) (Fei et al., 2020). Although the primary function of VP1 is to protect viral nucleic acids, it also participates directly in the viral infection process (Procházková et al., 2018). While previous studies have confirmed that structural proteins of the *Ralstonia solanacearum* genus play an important role in viral entry and replication (Dutta and Smith, 2023; Huang et al., 2020), the specific molecular mechanisms by which VP1 specifically interacts with host factors to influence viral replication remain unclear. Currently, the most widely used methods to investigate protein interactions include membrane yeast two-hybrid (MbY2H), surface plasmon resonance, co-immunoprecipitation (Co-IP), and glutathione-S-transferase (GST) pull-down assays (Douzi, 2017; Jia et al., 2020; Gnanasekaran and Pappu, 2023; Ramani et al., 2012). Among these, the MbY2H system is considered particularly effective for studying interactions involving membrane proteins. Unlike the classical Y2H system, which is restricted to analyzing nuclear interactions. MbY2H system is based on ubiquitin complementation. This allows the detection of interactions between membrane and cytoplasmic proteins in their native membrane environment (Yao et al., 2024).

Therefore, this study aimed to use the MbY2H system to screen a complementary DNA (cDNA) library for host membrane proteins that interact with VP1. Among the candidate proteins identified, those containing neuropeptide capa receptor (NCR)-like sequences were further validated for their interaction with VP1. Based on these findings, we used a bee pupa infection model to explore the role of these host-interacting proteins in the viral infection process. Our results elucidate the biological function of the host protein NCR-like protein during DWV infection, thereby contributing to an understanding of the pathogenic mechanisms of DWV.

## 2 Materials and methods

### 2.1 Viruses, plasmids, yeast strains, and clinical samples

Purified DWV, human embryonic kidney (HEK293T) cells, the Western honeybee membrane protein yeast cDNA library, and healthy Western honeybee pupae were all provided and maintained by the Laboratory Animal Center of Jinzhou Medical University. Plasmids pET-28a and pGEX-6P-1, alongside *Escherichia. coli* BL21(DE3) competent cells were purchased from TransGen Biotech (Beijing, China). The pTT5 plasmid, NMY32 yeast strain, and yeast vector plasmids were purchased from Haike Biotechnology Co., Ltd. (Shanghai, China).

### 2.2 Construction of relevant vectors

Primers were designed based on the gene sequences of DWV VP1 (GenBank ID: No. MF770715.1) and NCR-like (GenBank ID: XM_026446382.1) (Table 1). These gene sequences were amplified by polymerase chain reaction (PCR) and cloned into their corresponding vectors, yielding the pBT3-STE-VP1, pET-28a-NCR-like, and pGEX-6P-1-VP1 plasmids. The recombinant plasmids were identified using restriction enzyme digestion. pBT3-STE-VP1 underwent single digestion with *SfiI* at 50 °C for 40 min. pET-28a-NCR-like was double-digested with *BamHI* and *EcoRI*. pGEX-6P-1-VP1 was double-digested with *BamHI* and *XhoI*, and pTT5-VP1-Strep and pTT5-NCR-like-His were double-digested with *HindIII* and *EcoRI*. All double digestions were carried out at 37 °C for 30 min. The digested fragments were visualized via 1% agarose gel electrophoresis. Plasmids showing the correct restriction patterns were submitted for sequencing (Sangon Biotech Co., Ltd., Shanghai, China).

**Table 1:** Primers.

Primer Name	Sequence(5′ to 3′)
DWV-VP1-F	5’-GCGGCCATTACGGCCATGGATAATCCTTCTTATCAACA-3′
DWV-VP1-R	5’-GCGGCCGTAATGGCCTTATTCTGGAATAGCTTCAATAA-3′
pET-28a-NCR-like-F	5’-GCGGATCCATGACCATCATCTACATGATCAT-3’
pET-28a-NCR-like-R	5’-GCGAATTCTTATTTGCAGCAGATGGTCTGTT-3’
pGEX-6P-1-VP1-F	5’-GCGGATCCATGGATAATCCTTCTTATCAACA-3’
pGEX-6P-1-VP1-R	5’-GCCTCGAGTTATTCTGGAATAGCTTCAATAA-3
NCR-like-F	5’-CGTTTACGCGCAGGAATCTG-3’
NCR-like-R	5’-CAGGCAACCAGACAGGATGT-3’
Defensin-1-F	5’-TGCGCTGCTAACTGTCTCAG-3’
Defensin-1-R	5’-AATGGCACTTAACCGAAACG-3’
Hymenoptaecin-F	5’-CTCTTCTGTGCCGTTGCATA-3’
Hymenoptaecin-R	5’-GCGTCTCCTGTCATTCCATT-3’
β-actin-F	5’-ATGCCAACACTGTCCTTTCTGG-3’
β-actin-R	5’-GACCCACCAATCCATACGGA-3’

### 2.3 Bait plasmid self-activation and functionality test

The pBT3-STE-VP1 and control plasmids were co-transformed into the NMY32 yeast strain and grown on SD/−Leu/−Trp (SD-TL), SD/-His/−Leu/−Trp (SD-TLH), and SD/−Ade/-His/−Leu/−Trp (SD-TLHA) deficiency plates (Table 2) for 4 days at 30 °C. Colony growth was observed to assess self-activation activity and functionality of the bait plasmid.

**Table 2:** Plating instructions for transformation reactions.

Reaction	AD plasmid	BD plasmid	Plate type	Remarks
1	pNubG-Fe65	pTSU2-APP	SD-TL, SD-TLH, SD-TLHA	Positive control
2	pPR3N	pTSU2-APP	SD-TL, SD-TLH, SD-TLHA	Negative control
3	pPR3N	pBT3STE-VP1	SD-TL, SD-TLH, SD-TLHA	Self-activation detection
4	pOST1-NubI	pBT3STE-VP1	SD-TL, SD-TLH, SD-TLHA	Functional test

### 2.4 Yeast two-hybrid screening with bait plasmid and cDNA library

The yeast membrane protein cDNA library and the pBT3-STE-VP1 bait plasmid were introduced into NMY32 cells and cultured at 30 °C for 4 days. Potentially positive clone colonies were selected on plates containing synthetic defined (SD) medium with Leu/Trp and Ade/-His/−Leu/−Trp. Following secondary screening on 80 mM 3-AT plates, strongly positive clones were tested for the LacZ reporter gene. Subsequently, plasmids from the positive clones were extracted for PCR amplification and sequencing. Candidate host proteins interacting with DWV VP1 were identified using the Basic Local Alignment Search Tool (BLAST). Based on the screening results, NCR-like, a member of the G-protein-coupled receptor (GPCR) family, was selected for further investigation. This protein not only serves as a functional receptor or co-receptor for various viruses but also plays a pivotal role in modulating host antiviral mechanisms (Tcherniuk et al., 2016; Li et al., 2025). Therefore, the NCR-like protein was selected for subsequent experiments.

### 2.5 GST pull-down assay

To evaluate the interaction between VP1 and NCR-like, a GST pull-down assay was performed. The pET-28a-NCR-like and pGEX-6P-1-VP1 plasmids were transformed into *E. coli* BL21 (DE3) competent cells, followed by induction, expression, and purification. Protein expression was induced with 0.5 mmol/L Isopropylthio-*β*-D-galactoside (IPTG) at 30 °C for 8 h. Subsequently, 5ug of purified His-NCR-like protein was added to this mixture, which was incubated overnight at 4 °C. The purified fusion protein pGEX-6P-1-VP1-GST (20 μg) was incubated with glutathione sepharose 4B beads (Coolab Technology Co., Ltd., Beijing) at 4 °C for 3 h. The agarose complex was eluted with elution buffer [50 mM Tris–HCl (pH 8.0), 10 mM reduced glutathione], separated, collected, centrifuged, and solubilized in 5 × protein loading buffer (Beyotime Biotechnology Co., Ltd., Shanghai, China). Finally, the collected proteins were analyzed using sodium dodecyl sulfate-polyacrylamide gel electrophoresis (SDS-PAGE) and western blotting. The proteins were separated by 10% SDS-PAGE at 120 V for 90 min. Each lane was loaded with 20 μg of total protein and 15 μL of eluted sample and then transferred onto polyvinylidene fluroride membranes (Millipore, Germany, IPVH00010) at 300 mA for 50 min. The membranes were blocked with 5% non-fat milk in Tris-buffered saline with Tween-20 (TBST) for 2 h at room temperature, and the incubated overnight at 4 °C with a mouse anti-His tag antibody (1:2000; Boaosen Biotechnology, Beijing, Cat# bsm-33004 M). After washing, a HRP-conjugated goat anti-mouse secondary antibody (1:5000; Boaosen Biotechnology, Cat# bs-34296G-HRP) was applied for 1 h at room temperature. Bands were detected using enhanced chemiluminescence (NCM Biotech, China, Cat# P10060).

### 2.6 Co-IP

Co-IP assays were performed further to confirm the interaction between VP1 and NCR-like *in vitro*. The pTT5-VP1-Strep and pTT5-NCR-like-His plasmids were separately transfected into HEK293T cells, or co-transfected together into HEK293T cells, using polyethylenimine transfection reagent (Qiyan Biotechnology, Beijing, China). Cells transfected with an empty vector served as the control group. After 48 h, cells were lysed using cell lysis buffer (50 mM Tris–HCl, [pH 7.4], 150 mM NaCl, 1 mM EDTA, 1% NP-40, containing protease inhibitor cocktail). The lysate was centrifuged at 12,000 rpm for 25 min at 4 °C, and the resulting supernatant was incubated overnight at 4 °C with mouse anti-Strep/mouse anti-His tag antibodies (1:5000; Boaosen Biotechnology, Beijing, China). Then, 30 μL of Protein A/G Plus Agarose (Santa Cruz Biotechnology, Santa Cruz, CA, United States) was added, and the mixture was incubated at 4 °C for 6 h. The beads were collected using a magnetic stand and washed three times with 500 μL of ice-cold PBS containing 0.1% Tween-20. The precipitated proteins were then analyzed by western blotting as described above. For each lane, 30 μg of input lysate and 15 μL of eluate were loaded. Reciprocal antibodies were used for detection: after anti-Strep IP, an anti-His antibody was applied; conversely, after anti-His IP, an anti-Strep antibody was applied.

### 2.7 Immunofluorescence and confocal microscopy

HEK293T cells were co-transfected with the pTT5-VP1-Strep and pTT5-NCR-like-His plasmids in a 24-well plate. One day after transfection, the cells were fixed with 4% paraformaldehyde (Lanjieke, Beijing, China) for 15 min, permeabilized with 0.5% Triton X-100 (GenScript Biotech, Shanghai, China) for 10 min, and then blocked with 5% bovine serum albumin (Solarbio, Beijing, China) for 2 h. A mixture of a mouse anti-Strep tag (1:400) and a rabbit anti-His tag antibody (1:400) was added to the cells and incubated overnight at 4 °C. After washing the cells three times in TBST, a mixture of goat anti-mouse and goat anti-rabbit fluorescent secondary antibodies (1:200) (Abcam, UK) was added, and incubated at room temperature in the dark for 2 h. The nuclei were then stained with DAPI (Solarbio, Beijing, China) for 5 min. The stained cells were viewed under a laser-scanning confocal microscope (Zeiss LSM 980, Carl Zeiss, Germany) using a 63 × oil-immersion objective. Images were acquired using Zen v3.10 software (Carl Zeiss) at a resolution of 1,024 × 1,024 pixels, with pinhole set to 1 Airy unit. The excitation/emission wavelengths were 488/510, 561/580, 405/450 nm for FITC, Cy3, and DAPI, respectively.

### 2.8 RNAi-mediated knockdown of the NCR-like gene and detection of AMP in honeybee pupae

A previous study, Liu et al. (2024) indicated that the insect GPCR protein family is essential for regulating antimicrobial peptide (AMP) expression. Therefore, RNAi experiments were performed to knock down the host NCR-like gene and investigate its effects on AMP production. The double-stranded RNA (dsRNA) targeting the NCR-like gene was designed and synthesized by Shanghai Zhisheng Yougu Biotechnology Co., Ltd. Thirty healthy 9-day-old Western honeybee pupae were randomly divided into two groups (*n* = 15): a dsNCR-like group and a control group. Pupae in the dsNCR-like group were injected with 0.8 μL of dsRNA solution targeting NCR-like, whereas purpae in the control group received the same volume of dsRNA targeting green fluorescent protein (GFP), administered via a microsyringe (Hamilton, Switzerland, Cat# 65457–01). At 24, 48, and 72 h post-injection, individual pupae were collected and homogenized in TRIzol reagent (Invitrogen, America). Total RNA was extracted according to the manufacturer’s instructions, and cDNA was synthesized using a reverse transcription kit (TransGen, Beijing). Using β-actin as an internal control, SYBR Green qPCR was used to detect NCR-like gene expression in the host at these three time points. Concurrently, the expression levels of the host AMPs Defensin-1 and Hymenoptaecin were detected 24 h after dsRNA injection. The amplification protocol was as follows: denaturation at 95 °C for 2 min, followed by 40 cycles of denaturation at 95 °C for 15 s, and extension at 60 °C for 1 min. Primer sequences are listed in Table 1. Relative expression levels were calculated using the 2^-ΔΔCt^ method.

### 2.9 Effect of NCR-like gene knockdown on DWV replication

To investigate the effect of NCR-like genes on DWV replication, a DWV-infected Western honeybee pupa model (Supplementary Figure S1) was used. The experimental animals were divided into a DWV group (injected with dsGPF + DWV) and a dsNCR-like group (injected with dsNCR-like + DWV) (*n* = 15). First, each group was injected with dsRNA targeting NCR-like or GFP. Twenty-four hours later, they were reinjected with 1.36 × 10^6^ copies/μL of purified DWV. Pupae that died within 48 h were removed, and the surviving ones were collected. Total RNA was extracted from the collected specimen and reverse-transcribed into cDNA. DWV copy numbers in pupae were determined by reverse transcriptase (RT)-qPCR. Concurrently, expression levels of the Defensin-1 and Hymenoptaecin genes were compared between the DWV and dsNCR-like groups.

### 2.10 Statistical methods

All data were analyzed using GraphPad Prism 10.0 software. T-tests were used to assess statistical significance in intergroup comparisons. Data are presented as “mean ± standard deviation (SD).” *p*-values < 0.05 and < 0.01 indicated significant and highly significant differences, respectively. In all figures, * indicates *p* < 0.05, ** indicates *p* < 0.01, and *** indicates *p* < 0.001.

## 3 Results

### 3.1 Construction of recombinant plasmids

**Figure 1:**
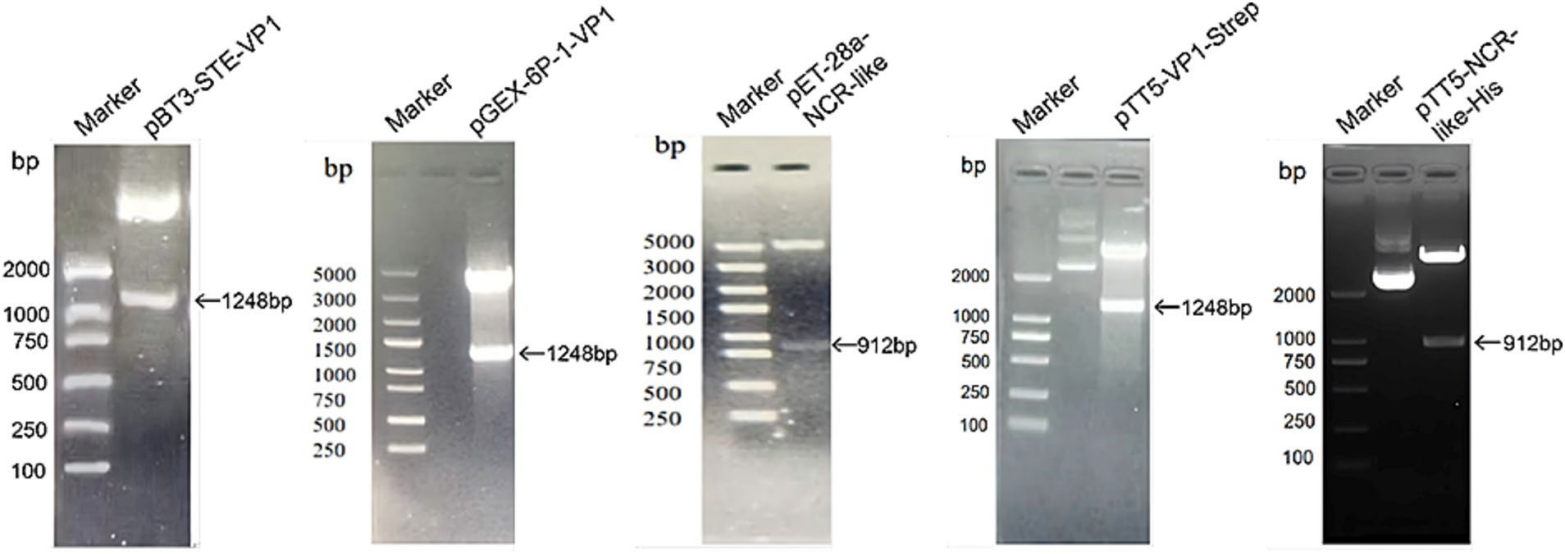
Construction of the pBT3-STE-VP1, pET-28a-NCR-like, pGEX-6P-1-VP1, pTT5-VP1-Strep, and pTT5-NCR-like-His plasmids. The pBT3-STE-VP1 plasmid was digested by the restriction endonuclease *SfiI*; The pET-28a-NCR-like plasmid was double-digested by the restriction endonucleases *BamHI* and *EcoRI*. The pGEX-6P-1-VP1 plasmid was double-digested by the restriction endonucleases *BamHI* and *XhoI*. The pTT5-VP1-Strep and pTT5-NCR-like-His plasmids were double-digested by the restriction endonuclease *HindIII* and *EcoRI*.

The positive recombinant plasmids pBT3-STE-VP1, pET-28a-NCR-like, pGEX-6P-1-VP1, pTT5-VP1-Strep, and pTT5-NCR-like-His were identified through restriction digestion and sequencing analysis, confirming that all recombinant plasmids had been successfully constructed (Figure 1). Meanwhile, to verify recombinant protein expression, the recombinant plasmids pGEX-6P-1-VP1 and pET-28a-NCR-like were transformed into *E. coli* competent cells, respectively, and expression was induced by IPTG. Subsequently, SDS-PAGE analysis was performed to examine protein expression. Under induction conditions of 30 °C and 0.5 mmol/L IPTG for 8 h, bands of approximately 70 and 39 kDa appeared in the precipitates, respectively (Supplementary Figure S2). The expressed proteins were then purified and analyzed by SDS-PAGE, revealing effective removal of contaminating proteins during purification.

### 3.2 Auto activation and function detection of the pBT3-STE-VP1 plasmid

**Figure 2:**
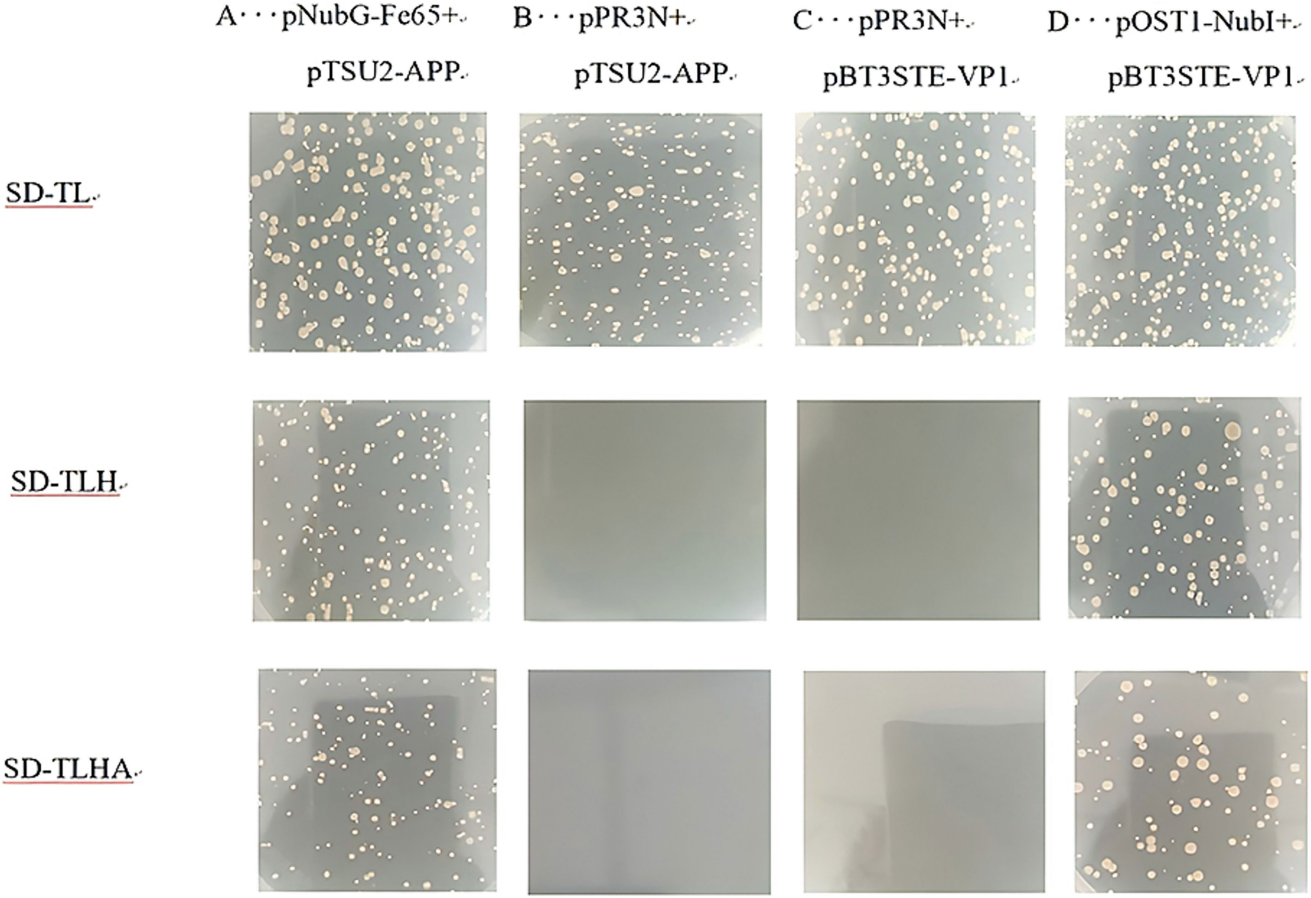
Results of autoactivation and function detection of the pBT3-STE-VP1 plasmid. **(A)** Positive control group: pNubG-Fe65 + pTSU2-APP. **(B)** Negative control group: pPR3N + pTSU2-APP. **(C)** Self-activation test group: pPR3N + pBT3-STE-VP1. **(D)** Functional test group: pOST1-NubI + pBT3-STE-VP1.

As shown in Figure 2, white colonies were observed on all type II yeast nutrient-deficient solid medium (SD-TL), type III (SD-TLH), and type IV (SD-TLHA) yeast nutrient-deficient solid plates in the positive control (pNubG-Fe65 + pTSU2-APP) and functional test groups (pOST1-NubI + pBT3-STE-VP1). However, these colonies were absent in the negative control (pPR3N + pTSU2-APP) and self-activation test groups (pPR3N + pBT3-STE-VP1) on the SD-TLH and SD-TLHA plates. These results demonstrate that the bait plasmid does not exhibit self-activation and functions normally in yeast cells, making it suitable for subsequent experiments.

### 3.3 Screening for DWV VP1-interacting host proteins

Using the MbY2H system with VP1 as bait, we screened the honeybee protein cDNA library and obtained 44 candidate positive yeast colonies that also activated the reporter gene LacZ (Supplementary Figure S3). After the positive yeast clones were subjected to plasmid extraction and PCR analysis, 31 candidates displayed amplified specific bands ranging from 500 to 2000 bp (Supplementary Figure S4). Using sequencing analysis, 22 interacting protein genes were identified (Supplementary Table S1). Following further analysis, the membrane protein NCR-like was selected for subsequent experiments.

### 3.4 GST pull-down assay

**Figure 3:**
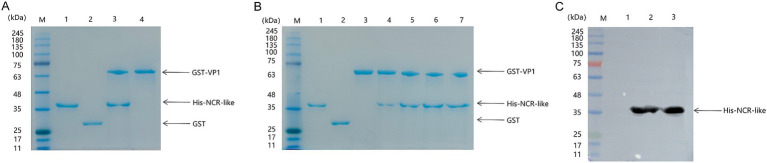
GST pull-down results. **(A)** M: Marker, 1: positive control (Input) His-NCR-like, 2: negative control GST tag, 3: experimental group (Pull-down) GST-VP1 + His-NCR-like, 4: GST-VP1 protein. **(B)** Concentration-dependent pull-down assay. A fixed amount of GST-VP1 (20 μg) was incubated with increasing amounts of purified His-NCR-like (5, 10, 20, and 40 μg). The amount of pulled-down His-NCR-like increased in a dose-dependent manner and reached saturation at approximately 20 μg. **(C)** M, Marker; 1, Negative control; 2, Positive control; 3, Experimental group. When detected using an anti-His-tag antibody, a band a band for the His-NCR-like protein appeared at approximately 39 kDa.

To verify the specific interaction between GST-VP1 and His-NCR-like protein, GST pull-down assays were performed using a single concentration (20 μg each) of both componets. The absence of binding in the negative control (GST tag alone) indicated that the interaction was protein-dependent. Subsequently, we performed additional GST pull-down experiments using a fixed amount of GST-VP1 (20 μg) and increasing concentrations of His-NCR-like (5, 10, 20, and 40 μg). SDS-PAGE analysis revealed that the amount of bound His-NCR-like (with a target band of approximately 39 kDa) increased in a dose-dependent manner with an increase in protein concentration, approaching saturation at approximately 20 μg (Figure 3). These results provide further confirmation that the interaction between VP1 and NCR-like is specific and concentration-dependent.

### 3.5 Co-IP assay

**Figure 4:**
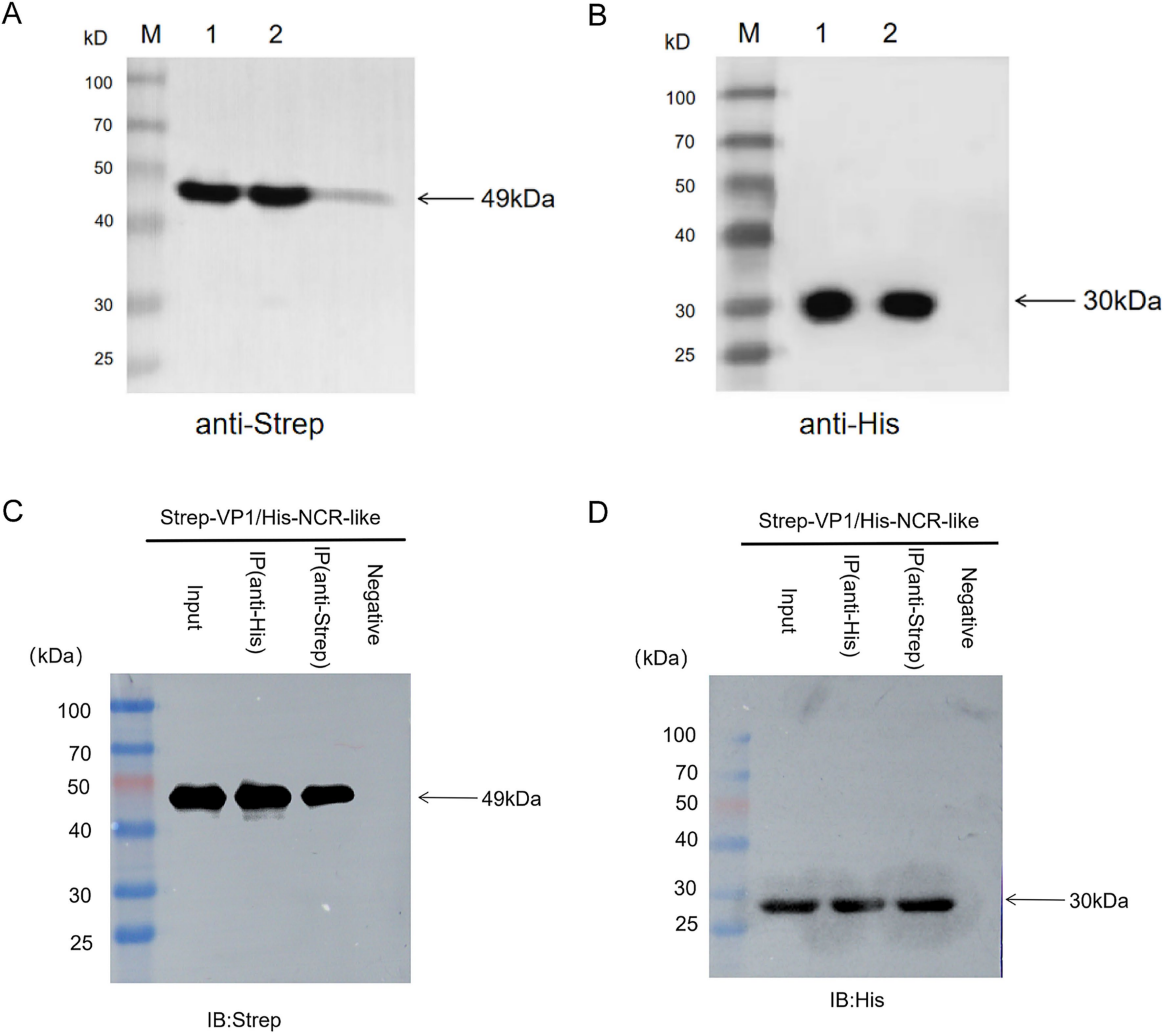
Co-IP analysis results of the interaction between VP1 and NCR-like. **(A,B)** The pTT5-VP1-Strep and pTT5-NCR-like-His plasmids were transfected into HEK293T cells to detect protein expression, respectively. M: protein marker; 1, 2: pTT5-VP1-Strep/pTT5-His. **(C,D)** In the recovered products, NCR-like protein bands were detected using anti-His antibody, and VP1 protein bands were detected using anti-Strep antibody. Negative controls were performed using cells transfected with VP1-Strep alone or NCR-like-His alone, respectively. No corresponding band was detected in the negative control.

To verify protein expression, HEK293T cells were separately transfected with the pTT5-VP1-Strep or pTT5-NCR-like-His plasmids. At 48 h post-transfection, the cells were collected and lysed. Western blot analysis confirmed the successful expression of both target proteins (Figure 4). Subsequently, to further confirm the interaction between VP1 and NCR-like in cells, a Co-IP assay was performed using proteins from co-transfected HEK293T cells. In the Co-IP experiments, NCR-like was precipitated using Strep as the bait, and VP1 was precipitated when His was used as the bait. HEK293T cells were transfected with empty vectors (pTT5-Strep or pTT5-His) alone were used as negative controls. Immunoprecipitation was subsequently performed using anti-Strep or anti-His antibodies, respectively. No specific bands corresponding to VP1 or NCR-like were detected in these control groups, confirming the specificity of the interactions (Figure 4). These results further support the interactions between NCR-like and VP1.

### 3.6 Immunofluorescence staining and confocal microscopy

**Figure 5:**
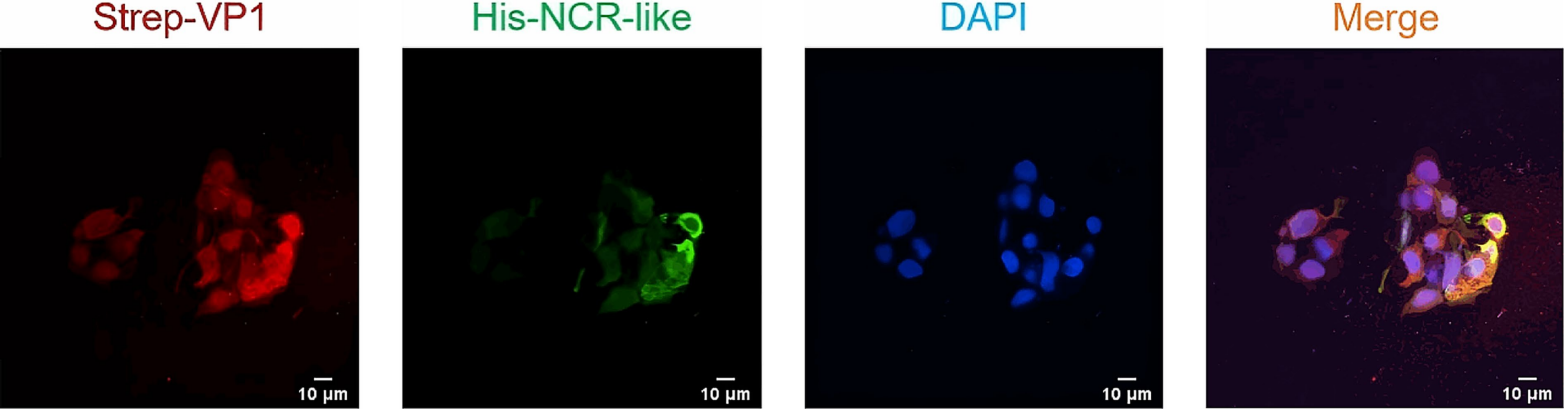
Colocalization of VP1 and NCR-like using immunofluorescence staining and confocal microscopy. HEK293T cells were co-transfected with pTT5-VP1-Strep and pTT5-NCR-like-His. VP1 was detected using mouse anti-Strep antibody, followed by Cy3-labeled goat anti-mouse secondary antibody (red). NCR-like was detected with rabbit anti-His antibody, followed by FITC-labeled goat anti-rabbit secondary antibody (green). Nuclei were stained with DAPI (blue). Scale bar: 10 μm. Magnification: 63 × oil objective. The merged image shows yellow regions (overlay of red and green), indicating colocalization of VP1 and NCR-like, predominantly in the cytoplasm and at the cell periphery. All panels show a single optical XY plane, not a Z-stack projection.

To determine the intracellular colocalization of VP1 and the membrane protein NCR-like, pTT5-NCR-like-His and pTT5-VP1-Strep were co-transfected into HEK293T cells. The results showed that Strep-VP1 and His-NCR-like proteins appeared to colocalize predominantly in the cytoplasm and at the cell periphery (Figure 5).

### 3.7 Effects of RNAi-mediated knockdown of the NCR-like gene on AMP expression

**Figure 6:**
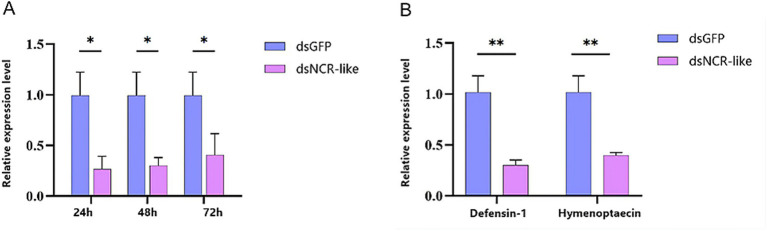
The dsGFP group comprises healthy bee pupae injected with dsGFP (serving as the negative control), and the dsNCR-like group comprises healthy bee pupae injected with dsNCR-like. **(A)** Detection of NCR-like gene expression levels at different time points (24, 48, and 72 h) following dsRNA injection. **(B)** Detection of AMP expression levels after NCR-like gene knockdown. Data are presented as mean ± SD, with differences analyzed using t-tests. *p < 0.05, **p < 0.01, ***p < 0.001.

To determine whether the NCR-like gene regulates the expression of immune effector AMPs, the NCR-like gene was knocked down in healthy bee pupae using RNAi. After injecting dsNCR-like into healthy bee pupae, NCR-like gene expression was analyzed at different time points. The results showed that, compared with the control group, NCR-like expression in the pupae was significantly reduced 24 h post-injection (*p* < 0.05), and this suppression lasted up to 72 h (Figure 6A). Therefore, 24 h post-dsRNA injection was designated as the time point at which the NCR-like gene was knocked down. One day after dsRNA injection, RT-qPCR analysis revealed that, compared with the control group injected with dsGFP, NCR-like gene knockdown resulted in a significant decrease in the relative mRNA expression levels of Defensin-1 and Hymenoptaecin (Figure 6B). These results indicate that NCR-like regulates the expression of Defensin-1 and Hymenoptaecin.

### 3.8 Effect of the NCR-like gene on viral replication

**Figure 7:**
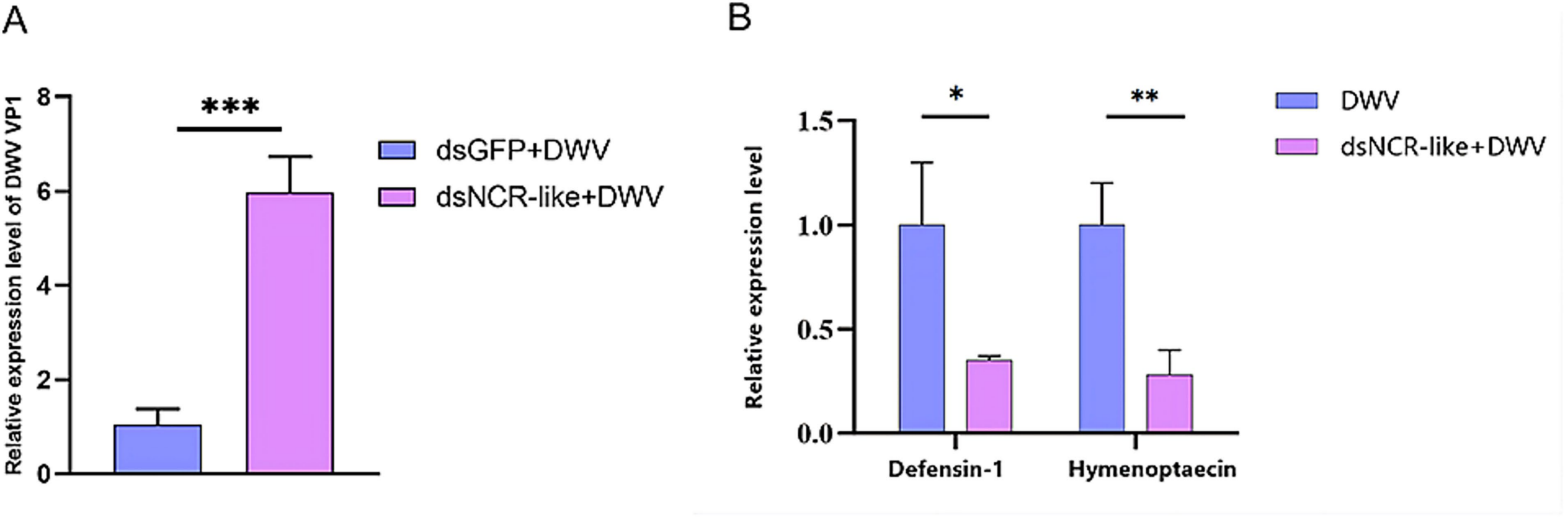
The DWV group comprises bee pupae inoculated with 1.36 × 10^6^ copies/μL of DWV. **(A)** Detection of DWV expression levels after NCR-like gene knockdown and virus inoculatio. **(B)** Detection of AMP expression levels after NCR-like gene knockdown and virus inoculation. Data are presented as mean ± SD, with differences analyzed using *t*-tests. *p < 0.05, **p < 0.01, ***p < 0.001.

To further investigate the role of the NCR-like gene in DWV replication within bees, a DWV-infected pupa model was established (more details in Supplementary Figure S1). The pupae in the dsNCR-like (dsRNA + DWV) and DWV (dsGFP + DWV) groups were pretreated with dsNCR-like and dsGFP, respectively, and then injected with purified DWV. The results showed that, compared with the group infected with DWV alone, the DWV copy number in the dsNCR-like + DWV group was approximately sixfold higher (*p* < 0.01) (Figure 7A) following NCR-like gene knockdown. Furthermore, the expression levels of Defensin-1 and Hymenoptaecin were further downregulated to 0.36 ± 0.01 and 0.46 ± 0.07, respectively (Figure 7B). These results suggest that the host NCR-like gene may modulate DWV replication, potentially through the regulation of specific immune effectors, such as Defensin-1 and Hymenoptaecin.

## 4 Discussion

Currently, DWV remains one of the most widespread and destructive honey bee pathogens, capable of inducing CCD and leading to the destruction of entire colony (Li, 2017; Škubník et al., 2017; Schläppi et al., 2019). Furrhermore, DWV exhibits a remarkable capacity for cross-species transmission, infecting bees within a single colony as well as across colonies through other species (Miles et al., 2023). Although VP1 is the largest structural protein of DWV and exhibits strong immunogenicity (Fei et al., 2020), its exact functional roles in host-virus interactions and pathogenesis remain uncharacterized.

In this study, yeast two-hybrid screen identified 44 positive clones, from which 22 host proteins were identified via sequencing and BLAST alignment (Bachler et al., 2025). Although the cDNA library was enriched for membrane protein-coding sequences, the identified candidates were functionally involved in diverse physiological processes, such as metabolism, immune regulation, and signal transduction. This diversity reflects the broad functional repertoire of membrane-associated proteins and their interaction partners, suggesting that DWV VP1 interacts with a diverse set of host proteins to exert a multifaceted role during viral infection. Among the candidates, NCR-like proteins in bees are membrane proteins belonging to the GPCR family (Reid et al., 2015). They perform diverse biological functions, including the regulation of insect immune responses at molecular and cellular levels (Urbanski and Rosinski, 2018). Studies have shown that during viral invasion, the viral first fuses with the host cell membrane (Song et al., 2026). NCR-like functions as a candidate receptor (Maginnis, 2023; Sandberg et al., 2024; Wang et al., 2020) or entry cofactor for DWV infection. Furthermore, NCR-like is necessary for inducing AMPs, thereby acting as a signaling regulator essential for mounting an effective antiviral immune response (Liu et al., 2024; Vannette et al., 2015). Therefore, the NCR-like protein was selected for further study.

In the present study, the interaction between VP1 and NCR-like was validated using multiple *in vitro* experiments. As bee-derived cell lines are currently unavailable, the mammalian cell line HEK293T was selected for Co-IP assays. To ensure the feasibility of the experiments, codon optimization was performed for both VP1 and NCR-like genes. Furthermore, confocal microscopy results revealed that VP1 and NCR-like colocalize in the cytoplasm and on the cell membrane, suggesting that their interaction primarily occurs in these regions.

Studies have shown that insect GPCR proteins play a key role in initiating and regulating innate immune signaling pathways (Kingsolver et al., 2013). They also exert antiviral functions by regulating immune effectors, including AMPs (Defensin-1 and Hymenoptaecin), via the Toll pathway (Guo et al., 2021). Defensin-1 and Hymenoptaecin were selected because they are prototypical effector molecules of the honeybee Toll pathway and have been reported to be regulated by GPCR signaling (Reboul and Ewbank, 2016). Their expression levels serve as reliable indicators of host immune status during viral infection. For example, bees fed jute nanocrystalline cellulose exhibit increased AMP expression, which can attenuate Israeli acute paralysis virus infection (Deng et al., 2023; EI-Seedi et al., 2020). The significant downregulation of Defensin-1 and Hymenoptaecin following NCR-like knockdown in healthy pupae suggests that this GPCR positively regulates key antimicrobial effectors. This immunomodulatory function appears to be biologically relevant during infection because the exacerbation of viral replication observed upon NCR-like silencing correlates strictly with the suppression of these AMPs. These findings suggest that DWV VP1 may exploit the NCR-like signaling axis to dampen the host’s humoral immunity, thereby creating a more permissive cellular environment for viral proliferation. Ideally, the effect of NCR-like knockdown on VP1 protein levels should directly demonstrate the functional relevance of this interaction. However, due to the current lack of a specific and sensitive anti-VP1 antibody for bee pupa lysates, we measured DWV RNA copy numbers as a standard indicator of viral replication. Future studies developing such antibodies will help further validate this mechanism. Nevertheless, the precise mechanism by which the DWV VP1 and NCR-like proteins activate the Toll pathway requires further investigation. Additionally, another limitation of this study is that while RNAi experiments targeted the NCR-like gene, overexpression of the NCR-like protein was not assessed, which should be addressed in future studies.

In summary, using a yeast two-hybrid system for membrane proteins, we identified NCR-like as a potential host membrane protein that interacts with DWV VP1. This interaction was validated by GST pull-down, Co-IP, and immunofluorescence experiments, providing strong evidence that the host NCR-like protein interacts with the DWV VP1 protein. Furthermore, the host NCR-like gene influences DWV replication by regulating the expression of immune effector AMPs. Collectively, these findings provide mechanistic insights into the interactions between DWV viral proteins and host proteins, as well as host immune defense mechanisms. In addition, this study provides a new strategy for investigating interactions between other bee viruses and their hosts.

## Data Availability

The datasets presented in this study can be found in online repositories. The names of the repository/repositories and accession number(s) can be found in the article/Supplementary material.

## References

[ref1] Bachler A. Walsh T. K. Rane R. V. Pandey G. (2025). Chimeric mis-annotations of genes remain pervasive in eukaryotic non-model organisms. BMC Genomics 26:630. doi: 10.1186/s12864-025-11765-w, 40597599 PMC12220653

[ref2] Chen G. Wu Y. Deng J. Wen Z. Wang S. Chen Y. . (2021). Seasonal variation of viral infections between the eastern honey bee (*Apis cerana*) and the western honey bee (*Apis mellifera*). Microbiology 10:e1162. doi: 10.1002/mbo3.1162, 33650796 PMC7862873

[ref3] De Miranda J. R. Genersch E. (2010). Deformed wing virus. J. Invertebr. Pathol. 103, S48–S61. doi: 10.1016/j.jip 19909976

[ref4] Deng Y. Yang X. Chen J. Yang S. Chi H. Chen C. . (2023). Jute (Corchorus olitoriusm L.) Nanocrystalline cellulose inhibits insect virus via gut microbiota and metabolism. ACS Nano 17, 21662–21677. doi: 10.1021/acsnano.3c06824, 37906569

[ref5] Douzi B. (2017). Protein-protein interactions: surface plasmon resonance. Methods Mol. Biol. 1615, 257–275. doi: 10.1007/978-1-4939-7033-9_21 28667619

[ref6] Dutta S. Smith M. D. (2023). Detection of protein-protein interactions utilizing the Split-ubiquitin membrane-based yeast two-hybrid system. Methods Mol. Biol. 2690, 37–57. doi: 10.1007/978-1-0716-3327-4_4, 37450135

[ref7] EI-Seedi H. Abd EI-Wahed A. Yosri N. Musharraf S. G. Chen L. Moustafa M. . (2020). Antimicrobial properties of *Apis mellifera*'s bee venom. Toxins (Basel). 12:451. doi: 10.3390/toxins12070451 32664544 PMC7404974

[ref9] Fei D. Guo Y. Fan Q. Li M. Sun L. Ma M. . (2020). Codon optimization, expression in Escherichia coli, and immunogenicity analysis of deformed wing virus (DWV) structural protein. PeerJ. 8:e8750. doi: 10.7717/peerj.8750, 32201647 PMC7071823

[ref10] Fei D. Guo Y. Fan Q. Wang H. Wu J. Li M. . (2019). Phylogenetic and recombination analyses of two deformed wing virus strains from different honeybee species in China. PeerJ. 7:e7214. doi: 10.7717/peerj.7214, 31293837 PMC6601602

[ref11] Genersch E. (2010). Honey bee pathology: current threats to honey bees and beekeeping. Appl. Microbiol. Biotechnol. 87, 87–97. doi: 10.1007/s00253-010-2573-8, 20401479

[ref12] Gnanasekaran P. Pappu H. R. (2023). Detection of protein-protein interactions using glutathione-S-transferase (GST) pull-down assay technique. Methods Mol. Biol. 2690, 111–115. doi: 10.1007/978-1-0716-3327-4_10, 37450141

[ref13] Guo Y. Zhang Z. Zhuang M. Wang L. Li K. Yao J. . (2021). Transcriptome profiling reveals a novel mechanism of antiviral immunity upon Sacbrood virus infection in honey bee larvae (*Apis cerana*). Front. Microbiol. 12:615893. doi: 10.3389/fmicb.2021.615893, 34149631 PMC8208235

[ref14] Huang S. Fei D. Ma Y. Wang C. Shi D. Liu K. . (2020). Identification of a novel host protein interacting with the structural protein VP2 of deformed wing virus by yeast two-hybrid screening. Virus Res. 286:198072. doi: 10.1016/j.virusres.2020.198072, 32659307

[ref15] Jia J. Jin J. Chen Q. Yuan Z. Li H. Bian J. . (2020). Eukaryotic expression, co-IP and MS identify BMPR-1B protein-protein interaction network. Biol. Res. 53:24. doi: 10.1186/s40659-020-00290-7, 32471519 PMC7257232

[ref16] Kingsolver M. B. Huang Z. Hardy R. W. (2013). Insect antiviral innate immunity: pathways, effectors, and connections. J. Mol. Biol. 425, 4921–4936. doi: 10.1016/j.jmb.2013.10.006, 24120681 PMC4007215

[ref17] Li W. S. (2017). Revisiting the mystery of the "CCD" phenomenon. China Bee J. 68, 59–60. doi: 10.3969/j.issn.0412-4367.2017.04.029

[ref18] Li S. Zhao J. Song Z. Zhang X. Lang B. Wang C. . (2025). Niacin, an active form of vitamin B3, exerts antiviral function by recruiting β-arrestin through GPR109A to activate the phosphorylation of ERK and STAT1 axis. J. Virol. 99:e0151925. doi: 10.1128/jvi.01519-25, 41159774 PMC12645984

[ref19] Liu J. Chen W. Situ J. Li J. Chen J. Lai M. . (2024). BmToll9-1 is a positive regulator of the immune response in the silkworm *Bombyx mori*. Insects. 15:643. doi: 10.3390/insects15090643, 39336611 PMC11432072

[ref20] Maginnis M. S. (2023). Β-Arrestins and G protein-coupled receptor kinases in viral entry: a graphical review. Cell. Signal. 102:110558. doi: 10.1016/j.cellsig.2022.110558, 36509265 PMC9811579

[ref21] Miles G. P. Liu X. F. Amiri E. Grodowitz M. J. Allen M. L. Chen J. (2023). Co-occurrence of wing deformity and impaired mobility of Alates with deformed wing virus in *Solenopsis invicta* Buren (Hymenoptera: Formicidae). Insects. 14:788. doi: 10.3390/insects14100788, 37887800 PMC10607916

[ref22] Nazzi F. Brown S. P. Annoscia D. Del Piccolo F. Di Prisco G. Varricchio P. . (2012). Synergistic parasite-pathogen interactions mediated by host immunity can drive the collapse of honeybee colonies. PLoS Pathog. 8:e1002735. doi: 10.1371/journal.ppat.1002735, 22719246 PMC3375299

[ref23] Organtini L. J. Shingler K. L. Ashley R. E. Capaldi E. A. Durrani K. Dryden K. A. . (2017). Honey bee deformed wing virus structures reveal that conformational changes accompany genome release. J. Virol. 91, e01795–e01716. doi: 10.1128/JVI.01795-16, 27852845 PMC5215330

[ref24] Parekh F. Daughenbaugh K. F. Flenniken M. L. (2022). Corrigendum: chemical stimulants and stressors impact the outcome of virus infection and immune gene expression in honey bees (*Apis mellifera*). Front. Immunol. 13:844882. doi: 10.3389/fimmu.2022.844882, 35309356 PMC8925646

[ref25] Procházková M. Füzik T. Škubník K. Moravcová J. Ubiparip Z. Přidal A. . (2018). Virion structure and genome delivery mechanism of sacbrood honeybee virus. Proc. Natl. Acad. Sci. USA 115, 7759–7764. doi: 10.1073/pnas.1722018115, 29987012 PMC6065027

[ref26] Ramani S. R. Tom I. Lewin-Koh N. Wranik B. Depalatis L. Zhang J. . (2012). A secreted protein microarray platform for extracellular protein interaction discovery. Anal. Biochem. 420, 127–138. doi: 10.1016/j.ab.2011.09.017, 21982860

[ref27] Reboul J. Ewbank J. J. (2016). GPCRs in invertebrate innate immunity. Biochem. Pharmacol. 114, 82–87. doi: 10.1016/j.bcp.2016.05.015, 27262554

[ref28] Reid C. R. Airo A. M. Hobman T. C. (2015). The virus-host interplay: biogenesis of +RNA replication complexes. Viruses 7, 4385–4413. doi: 10.3390/v7082825, 26287230 PMC4576186

[ref29] Sandberg A. L. Bond A. C. S. Bennett L. J. Craig S. E. Winski D. P. Kirkby L. C. . (2024). GPCR inhibitors have antiviral properties against JC polyomavirus infection. Viruses 16:1559. doi: 10.3390/v16101559, 39459893 PMC11512265

[ref30] Schläppi D. Lattrell P. Yañez O. Chejanovsky N. Neumann P. (2019). Foodborne transmission of deformed wing virus to ants (*Myrmica rubra*). Insects. 10:394. doi: 10.3390/insects10110394, 31703426 PMC6920936

[ref31] Škubník K. Nováček J. Füzik T. Přidal A. Paxton R. J. Plevka P. (2017). Structure of deformed wing virus, a major honey bee pathogen. Proc. Natl. Acad. Sci. USA 114, 3210–3215. doi: 10.1073/pnas.1615695114, 28270616 PMC5373406

[ref32] Song S. Li S. Luo Y. Wei D. Shang J. Wu H. . (2026). Dual disruption of the immune cytokine spätzle facilitates fungal infection of diverse insect hosts. Adv Sci (Weinh). 13:e13075. doi: 10.1002/advs.202513075, 41114474 PMC12767022

[ref33] Sui J. C. Yu H. S. Dai J. Y. Wang X. H. Zheng Y. Yang Q. . (2015). RT-PCR detection of deformed wing virus in honeybees and sequence analysis of capsid protein VP1 gene. Heilongjiang Anim. Sci. Vet. Med. 23, 157–159. doi: 10.13881/j.cnki.hljxmsy.2015.2083

[ref34] Sun L. Li M. Ma Y. Huang S. Ma M. Fei D. (2023). Interaction between the VP2 protein of deformed wing virus and host snapin protein and its effect on viral replication. Front. Microbiol. 14:1096306. doi: 10.3389/fmicb.2023.1096306, 36846748 PMC9945523

[ref35] Tcherniuk S. Cenac N. Comte M. Frouard J. Errazuriz-Cerda E. Galabov A. . (2016). Formyl Peptide Receptor 2 Plays a Deleterious Role During Influenza A Virus Infections. J Infect Dis. 24, 237–47. doi: 10.1093/infdis/jiw127 27034344

[ref36] Urbanski A. Rosinski G. (2018). Role of neuropeptides in the regulation of the insect immune system—current knowledge and perspectives. Curr. Protein Pept. Sci. 19, 1201–1213. doi: 10.2174/1389203719666180809113706, 30091409

[ref37] Vannette R. L. Mohamed A. Johnson B. R. (2015). Forager bees (*Apis mellifera*) highly express immune and detoxification genes in tissues associated with nectar processing. Sci. Rep. 5:16224. doi: 10.1038/srep16224, 26549293 PMC4637902

[ref38] Wang G. Jiang L. Wang J. Zhang J. Kong F. Li Q. . (2020). The G protein-coupled receptor FFAR2 promotes internalization during influenza a virus entry. J. Virol. 94, e01707–e01719. doi: 10.1128/JVI.01707-19, 31694949 PMC6955252

[ref39] Yao Z. L. Chang Z. Y. Gong W. Song T. T. Wang Z. X. Zhang Y. N. . (2024). Identification of key ubiquitin-conjugating enzymes during chicken PGCs formation based on yeast two-hybrid library. China Poult. 46, 15–22. doi: 10.16372/j.issn.1004-6364.2024.03.003

