# Supplementary material for: Screening and functional analysis of a host membrane protein interacting with the structural protein VP1 of deformed wing virus
Source: Front Microbiol. 2026 Aug 5;17:1860293. doi: 10.3389/fmicb.2026.1860293 (PMC13486166; doi:10.3389/fmicb.2026.1860293)
Supplement: Supplementary file 1 [file Table_1.DOCX]

****Supplementary Material****

**1. Results of the establishment of the bee pupa infection model**

**The clinical phenotypes of the pupae were observed, and the results are shown in Figure S1(A). Compared with the control group, significant differences were observed in pupae inoculated with 1.36 × 10⁶ copies/μL DWV at 48 h post-inoculation, including body surface melanization and pronounced abdominal atrophy. To determine the DWV copy number in infected pupae, RT-qPCR was performed, and the results are presented in Figure S1(B). The viral copy number reached approximately 10⁸–10⁹ copies/μL at 48 h post-inoculation with 1.36 × 10⁶ copies/μL DWV. Hematoxylin and eosin (H&E) staining of pathological tissue sections (Figure S1(C)) revealed that, compared with the control group, brain tissue of pupae inoculated with 1.36 × 10⁶ copies/μL DWV for 48 h exhibited tissue swelling, obvious local atrophy, widened perivascular spaces, acinar atrophy, and the presence of tissue debris. Based on these findings, inoculation with 1.36 × 10⁶ copies/μL DWV for 48 h was selected for subsequent experiments, which met the evaluation criteria for the established infection model in this study.**

Figure S1: Results of DWV challenge in bee pupae.(A) Clinical observations of pupae after DWV inoculation.(B) Detection of viral copy numbers.(C) Hematoxylin and eosin (H&E) staining results: (a) control group; (b) 1.36 × 10⁵ copies/μL DWV, 48 h group; (c) 1.36 × 10⁶ copies/μL DWV, 48 h group; (d) 1.36 × 10⁷ copies/μL DWV, 48 h group.

2.Expression and Purification of Recombinant VP1 and NCR-like Proteins

The recombinant plasmids pGEX-6P-1-VP1 and pET-28a-NCR-like were separately transformed into *E. colicompetent* cells and induced with IPTG. The expression profiles were subsequently analyzed by SDS-PAGE. As shown in Supplementary Figure S4(A) and (B), induction at 30°C with 0.5 mmol/L IPTG for 8h resulted in distinct bands at 70 kDa and 39 kDa, respectively. Following purification, the samples were analyzed again by SDS-PAGE. As shown in Supplementary Figure S4(C), contaminating proteins were effectively removed during the purification process.

Figure S2: Prokaryotic expression and purification of recombinant VP1 and NCR-like proteins.(A) Lanes 1–3 show the pellet (insoluble fraction), and lanes 4–6 show the supernatant (soluble fraction) under corresponding IPTG (0.1, 0.25 and 0.5mmol/L) concentrations. Lane 7 represents the empty vector control. (B) Lanes 1–3 show the pellet, lanes 4–6 show the supernatant, and lane 7 represents the empty vector control. (C) M: Protein Marker; Lanes 1–2: Purified GST-VP1 recombinant protein. (D) M: Protein Marker; Lane 1: Purified His-NCR-like recombinant protein. Both target proteins were successfully enriched with effective removal of contaminating proteins.

**3.Results of LacZ reporter gene detection.**

**Semi-quantitative β-galactosidase activity assays were performed on the 44 initial positive yeast clones, and as shown in Figure S2, all of them passed the LacZ reporter gene detection.**

**Figure S3 β-Galactosidase activity results of positive clones. PC: positive control; NC: negative control**

**4.PCR detection of strongly positive clones**

**The 44 positive clones that passed the above screening and detection were cultured in SD-TL liquid medium, and the positive yeast plasmids were extracted. These yeast plasmids were then transformed into competent cells, and the plasmids were re-extracted for PCR identification. The results are shown in Figure S3. A total of 31 plasmids exhibited specific bands between 500–2000 bp, with the following numbers: 1, 5, 15, 16, 19, 20, 23, 27, 28, 39, 45, 52, 112, 114, 115, 116, 117, 118, 119, 125, 127, 130, 136, 137, 151, 152, 162, 163, 164, 185, 189.**

Figure S4 PCR identification of plasmids.

5.Subcellular localization and co-localization analysis of VP1 and NCR-like in HEK293T cells.

To address the concern regarding the subcellular compartment assignment (cytoplasm and cell membrane), we have performed additional colocalization analyses. We merged the single-channel images of Strep-VP1 and DAPI, as well as His-NCR-like and DAPI. The results clearly show that the red signal (VP1) and green signal (NCR-like) are distributed in the perinuclear region and along the cell periphery, distinct from the blue nuclear stain. This morphological evidence strongly supports that the co-localization occurs in the cytoplasm and at the cell periphery.

Figure S5. Subcellular localization and co-localization analysis of VP1 and NCR-like in HEK293T cells.

6. Figure S6 provides the original scans of the GST pull-down assays, corresponding to the cropped data presented in Figure 3 (concentration gradient and interaction validation).

Figure S6. Original, uncropped gel images of the GST pull-down assays.

7. Figure S7 provides the original scans of the Co-IP assays from HEK293T cells, corresponding to Figure 4. These raw data files clearly show the intact gel backgrounds, marker positions, and the specific bands discussed in the manuscript, ensuring the transparency and reproducibility of our results.

Figure S7. Original, uncropped gel images of the Co-IP assays.

8. To verify that the negative control samples were properly loaded and transferred in Figure 4, a parallel Western blot was performed using the identical batch of samples under the same experimental conditions. Due to the minimal molecular weight difference between the target protein and GAPDH, a sister membrane derived from the same gel run was probed with an anti-GAPDH antibody (Beyotime, Cat# AF1186). Lysates were quantified by BCA assay (20 µg per lane) prior to loading. Comparable GAPDH signals across all lanes, including the negative controls, confirm consistent protein loading and transfer efficiency, supporting the specificity of the Co-immunoprecipitation results presented in Figure 4.

Figure S8. Validation of sample loading by GAPDH detection.

**9. Analysis of sequencing results**

**The 31 plasmids described above were sequenced, and the obtained sequences were subjected to BLAST alignment analysis against the NCBI database. The sequencing analysis results are presented in Table S1. A total of 22 genes encoding interacting proteins were identified. Among these, the sole membrane protein, NCR-like, was selected for subsequent experiments.**

Table S1 Analysis of sequencing results

| No. | Positive clone ID | BLAST sequence analysis | | |
| --- | --- | --- | --- | --- |
|  |  | Gen Bank | English name | Chinese name |
| 1 | 5 | XM_026446382.1 | *Apis mellifera* capa receptor-like GPCR | 意大利蜜蜂capa受体GPCR |
| 2 | 19、20、45、115、117 | XM_001121282.5 | *Apis mellifera* 60S ribosomal protein L37a | 意大利蜜蜂60S核糖体蛋白L37a |
| 3 | 16、163 | NM_001011601.1 | *Apis mellifera* major royal jelly protein 3 | 意大利蜜蜂主要蜂王浆蛋白3 |
| 4 | 23 | [NM_001011583.2](https://www.ncbi.nlm.nih.gov/nucleotide/NM_001011583.2?report=genbank&log$=nucltop&blast_rank=6&RID=1ASG09YJ016) | *Apis mellifera* chemosensory protein 3 (CSP3) | 意大利蜜蜂化学感受蛋白3（CSP3） |
| 5 | 136 | AP018435.1 | *Apis mellifera* mitochondrial DNA | 意大利蜜蜂线粒体DNA |
| 6 | 39、164 | XM_006572192.3 | *Apis mellifera* 60S ribosomal protein L12 | 意大利蜜蜂60S核糖体蛋白L12 |
| 7 | 189 | [XM_006564701.3](https://www.ncbi.nlm.nih.gov/nucleotide/XM_006564701.3?report=genbank&log$=nuclalign&blast_rank=4&RID=1AUNNAE2014) | *Apis mellifera* probable salivary secreted peptide | 意大利蜜蜂唾液分泌肽 |
| 8 | 137 | [XM_006572191.3](https://www.ncbi.nlm.nih.gov/nucleotide/XM_006572191.3?report=genbank&log$=nuclalign&blast_rank=3&RID=1AV9J84R014) | *Apis mellifera* 60S ribosomal protein L12 | 意大利蜜蜂60S核糖体蛋白L12 |
| 9 | 185 | [XM_017059853.1](https://www.ncbi.nlm.nih.gov/nucleotide/XM_017059853.1?report=genbank&log$=nuclalign&blast_rank=1&RID=D8RAZG55014) | *Apis mellifera* major royal jelly protein 2 | 意大利蜜蜂主要蜂王浆蛋白2 |
| 10 | 130 | [XM_001121746.5](https://www.ncbi.nlm.nih.gov/nucleotide/XM_001121746.5?report=genbank&log$=nuclalign&blast_rank=3&RID=1AVDR4S0014) | *Apis mellifera* neurofilament heavy polypeptide | 意大利蜜蜂神经丝重多肽 |
| 11 | 27 | XM_016917951.1 | *Apis mellifera* early nodulin-75 | 意大利蜜蜂早期结瘤素-75 |
| 12 | 112 | [XM_394993.7](https://www.ncbi.nlm.nih.gov/nucleotide/XM_394993.7?report=genbank&log$=nuclalign&blast_rank=2&RID=1AVJES7R014) | *Apis mellifera* proteasome subunit beta type-4 | 意大利蜜蜂蛋白酶体β -4型亚基 |
| 13 | 114 | [XM_392518.7](https://www.ncbi.nlm.nih.gov/nucleotide/XM_392518.7?report=genbank&log$=nuclalign&blast_rank=3&RID=1AVMJVW3014) | *Apis mellifera* proteasome subunit alpha type-3 | 意大利蜜蜂蛋白酶体α -3型亚基 |
| 14 | 119 | [XM_624279.6](https://www.ncbi.nlm.nih.gov/nucleotide/XM_624279.6?report=genbank&log$=nuclalign&blast_rank=3&RID=1AVPGTJY014) | *Apis mellifera* prefoldin subunit 3 | 意大利蜜蜂前折叠亚基3 |
| 15 | 125 | [NM_001011574.1](https://www.ncbi.nlm.nih.gov/nucleotide/NM_001011574.1?report=genbank&log$=nuclalign&blast_rank=4&RID=1AVSK067016) | *Apis mellifera* glucose dehydrogenase | 意大利蜜蜂葡萄糖脱氢酶 |
| 16 | 127 | XM_006558669.3 | *Apis mellifera* transcriptional regulator ATRX homolog | 意大利蜜蜂转录调节剂ATRX同源物 |
| 17 | 151 | [XM_006557645.3](https://www.ncbi.nlm.nih.gov/nucleotide/XM_006557645.3?report=genbank&log$=nuclalign&blast_rank=5&RID=1AVY8M74016) | *Apis mellifera* 60S ribosomal protein L11 | 意大利蜜蜂60S核糖体蛋白L11 |
| 18 | 52 | [XM_016911957.2](https://www.ncbi.nlm.nih.gov/nucleotide/XM_016911957.2?report=genbank&log$=nuclalign&blast_rank=2&RID=1AW2027R016) | *Apis mellifera* 60S ribosomal protein L7 | 意大利蜜蜂60S核糖体蛋白L7 |
| 19 | 118 | XM_006563359.3 | *Apis mellifera* chymotrypsin inhibitor | 意大利蜜蜂凝乳胰蛋白酶抑制剂 |
| 20 | 1 | [XM_026444626.1](https://www.ncbi.nlm.nih.gov/nucleotide/XM_026444626.1?report=genbank&log$=nuclalign&blast_rank=5&RID=1AW3Y19X016) | *Apis mellifera* enolase | 意大利蜜蜂烯醇酶 |
| 21 | 15、28 | [XM_026443036.1](https://www.ncbi.nlm.nih.gov/nucleotide/XM_026443036.1?report=genbank&log$=nuclalign&blast_rank=5&RID=1AW96V5K014)L | *Apis mellifera* uncharacterized | 意大利蜜蜂推测未定性的蛋白 |
| 22 | 116、152、162 | XM_026440041.1 | *Apis mellifera* uncharacterized | 意大利蜜蜂推测未定性的蛋白 |
